# Preserving Differential Privacy for Similarity Measurement in Smart Environments

**DOI:** 10.1155/2014/581426

**Published:** 2014-07-15

**Authors:** Kok-Seng Wong, Myung Ho Kim

**Affiliations:** School of Computer Science and Engineering, Soongsil University, Information Science Building, Sangdo-dong, Dongjak-gu, Seoul 156-743, Republic of Korea

## Abstract

Advances in both sensor technologies and network infrastructures have encouraged the development of smart environments to enhance people's life and living styles. However, collecting and storing user's data in the smart environments pose severe privacy concerns because these data may contain sensitive information about the subject. Hence, privacy protection is now an emerging issue that we need to consider especially when data sharing is essential for analysis purpose. In this paper, we consider the case where two agents in the smart environment want to measure the similarity of their collected or stored data. We use similarity coefficient function ($F_{SC}$) as the measurement metric for the comparison with differential privacy model. Unlike the existing solutions, our protocol can facilitate more than one request to compute $F_{SC}$ without modifying the protocol. Our solution ensures privacy protection for both the inputs and the computed $F_{SC}$ results.

## 1. Introduction

Advances in both sensor technologies and network infrastructures have encouraged the growth and the development of smart environments. The concept of smart environments is to promote the ideas of small world with great deal of different smart devices such as sensors, microcontrollers, handheld devices, and computers that connected via wired or wireless networks [1]. These smart devices can automatically collect real-time data from the users without human-to-human or human-to-computer interaction. Note that smart devices can collect large amounts of personal data when the users are operating and interacting with the environment. The organization and exploration of these heterogeneous personal data require intelligent software agents (hereafter we will refer to them as agents) to do the analysis in order to trigger actions for the environment. A study of the exploration of personal data has been conducted in [2].

There are many smart spaces (e.g., smart home, smart building, and smart office) which have been proposed and developed in the past few years to enhance a person's environment and way of life. For example, smart homes for ubiquitous healthcare [3] can support patients who live independently at home by providing health monitoring and remote assistance [4]. Smart office can adapt itself to the user needs and hence release the users from their routine tasks [5]. In such environment, office workers can communicate, collaborate, and work in a new and more efficient way.

Along with the potential benefits offered, the usage of smart environment also raises some security and privacy concerns to the data owners. Since a large amount of user's data is captured and possibly stored, issues arise relating to the storage and usage of sensitive data. In the existing implementations, there is no clear privacy protection in place. This may cause the users feel uncomfortable to work or stay in the smart environments. Therefore, data privacy is one of the main challenges for acceptance and adoption of smart environments.

The data privacy concern arising in the smart environments is mainly about the usage of the collected data. The intelligent software agents analyze the collected data to understand the changes of the environment and perform activity prediction. Some of the data collected from the users may be sensitive and, hence, the access control to share those data is becoming an important task. In a multiagent smart environment, two or more agents may concurrently (or within a given period) collect data from the same user. A wide range of data analysis operations entails a similarity measurement between datasets collected. Based on the analysis results, the smart environments can improve the experience of their inhabitants by adapting the behavior of the users and other conditions in the environment.

When users (or agents) wish to compare datasets collected with other parties, a secure mechanism must be available to facilitate the computation in a secure manner. Assume that two parties would like to find the similarity between their collected datasets. We can utilize a measurement metric such as similarity coefficient for the comparison.

Similarity coefficient ($F_{SC}$) is a function used to study the coexistence of objects and the similarity of the objects. Finding similarities between two datasets is an important task in many research areas. The output from the comparison can be involved in such contexts as the study of the coexistence of species and the similarity of sampling sites [6, 7] (in the context of ecological and biogeographical research), as the matching of two given DNA sequences [8], or as the assignment of a set of observations into subsets called clusters [9] (in the clustering application). In the privacy preserving data mining (PPDM) applications such as clustering [9, 10], the similarity coefficient is used to assign a set of observations or data into subsets called clusters. Recently, similarity coefficient has also been applied in biometric areas to solve identification problems such as iris and fingerprint recognition [11].

### 1.1. Motivation

Advances in data collection technologies have led to an increasing number of data collected and stored in smart environments. In the early age, collected data were generally without considering security and privacy issues. Therefore, previously stored data may contain a vast amount of sensitive information. These data are important for the analysis purpose and for the comparison with the newly collected data in order to trigger accurate activity for the changing of the environment. Recent discussions about user's data privacy with respect to the data collected in the smart environment have shown that the public gradually realizes that this may have a long-term impact on their everyday life.

Let us consider a practical scenario where two agents (each embedded with a sensor) would like to analyze and extract useful information from the datasets they collected from the users. To improve the performance and accuracy of the changing condition in the environment, data from the same (or different) subject must be gathered and used for the analysis. These analyses require collaboration between agents and sharing of data collected by each sensor. However, the release and sharing of sensitive information raises some privacy concerns for the users.

In a context-sensitive environment, access to a resource requires the collection of confidential information. For instance, if the location of a person is used to grant access to resources such as printer and projector, the information about the acceptance or rejection of using a device will violate the person's privacy [12]. Consequently, privacy concerns arise in terms of how to control the sharing of sensitive information with other users or agents.

### 1.2. Problem Statement

In this paper, we will consider the comparison of both data types (old and newly collected data) for the similarity measurement. We define the problem in this paper as follows: let *X* = {*x*_1_, *x*_2_,…, *x*_*n*_} and *Y* = {*y*_1_, *y*_2_,…, *y*_*n*_} be two binary datasets belonging to two agents (a requestor and a supporter, resp.). We assume that the requestor wants to measure the similarity between *X* and *Y* without revealing *X* to the supporter. At the same time, the supporter is willing to participate if (1)  *Y*  will not be revealed to the requestor and (2) no extra information can be derived from the final output.

Since the same datasets may be used for several similarity measurements, we design our protocol to facilitate more than one computation (without modifying the protocol). To support multiple similarity coefficients, we utilize a semitrusted anonymizer in our protocol to answer the requests from the requestor.

The execution of our protocol should preserve a number of fundamental security properties as described in [13]. In particular, all players must ensure that no extra information will be revealed other than the computed output (privacy is protected) and the output of the protocol is according to the prescribed functionality (correctness is guaranteed). We require all computations in our protocol to be performed in an encrypted form by utilizing a semantically secure homomorphic cryptosystem in our protocol design. The details of the homomorphic cryptosystem will be discussed in Section 3.1.

### 1.3. Organization of the Paper

This paper is organized as follows.  Section 2 introduces the background for this research and discusses related works in the literature.  Section 3 describes the technical preliminaries of our work, followed by the details of our private similarity coefficients computation protocol in Section 4. The analysis and discussion of our protocol are presented in Section 5 and our conclusion is presented in Section 6.

## 2. Background and Related Work

### 2.1. Similarity Coefficients

Binary data is a representation of presence or absence of an attribute in the given objects. The value “1” is used to show the presence of the attribute while “0” is used to represent the absence of the attribute. Hence, a binary dataset is composed of a series of strings with “1” and “0.”

Let *X* = {*x*_*i*_∣*i* = 1,2,…, *n*} and *Y* = {*y*_*i*_∣*i* = 1,2,…, *n*} be two binary datasets, where *x*_*i*_, *y*_*i*_ ∈ {0, 1} and *C*_*XY*_ = {(*x*_*i*_, *y*_*i*_)∣*i* = 1,2,…, *n*}. We further specify the following summation variables.

- *a* is the number of (*x*_*i*_, *y*_*i*_) = (1,1) in *C*_*XY*_.
- *b* is the number of (*x*_*i*_, *y*_*i*_) = (1,0) in *C*_*XY*_.
- *c* is the number of (*x*_*i*_, *y*_*i*_) = (0,1) in *C*_*XY*_.
- *d* is the number of (*x*_*i*_, *y*_*i*_) = (0,0) in *C*_*XY*_.

In the relevant literature [14, 15], *a* is known as “positive match,” *b* and *c* are known as “mismatch,” and *d* is referred to as “negative match.”

The computation of similarity coefficient $F_{SC}$ is based on the summation variables. A large number of $F_{SC}$ have been proposed in the literature. Similarity coefficient choice is based on some criterion. An important consideration is the inclusion or exclusion of negative match *d* in the computation. For some data, the absence of an element in both objects would indicate similarity, but, in certain cases, this might not be true. Hence, we can divide the similarity coefficients into two types.

The first type takes into consideration negative matches. For example, Russell and Rao [16] introduced the similarity coefficient of this type that can be expressed as follows:

(1) $$\begin{array}{c} \frac{a}{a+b+c+d}. \end{array}$$

This similarity coefficient represents the proportion of positive matches in the dataset. Note that the denominator in (1) is actually the size of the dataset, *n*.

In the second type, we do not consider negative matches in the computation. For example, the Jaccard coefficient [17] can be calculated as follows:

(2) $$\begin{array}{c} \frac{a}{a+b+c}. \end{array}$$

As shown in (2), the Jaccard coefficient is independent of the summation variable *d*.

In the asymmetric type of binary data, the positive matches are usually more significant than the negative matches [18, 19]. However, the inclusion or exclusion of negative matches in the similarity coefficients is still an ongoing issue in many research areas [14, 20]. We refer readers to [21] for a comprehensive similarity coefficients list (the authors compiled a list of 76 binary similarity coefficients).

In this paper, we particularly consider the similarity coefficients for binary data, but with the correct size of each summation variable in Section 2.1, the agent is able to compute dissimilarity coefficients of two datasets (i.e., *X* and *Y*). We do not discuss further dissimilarity coefficients in this paper, but we would like to stress that our protocol is also applied to dissimilarity coefficients computation.

### 2.2. Related Work

Data privacy protection is still a major concern in smart environments, although there have been efforts to protect personal information of the users by using mobile agents [22] and deploying security framework [23] and context-based solutions (e.g., context-sensitive services [12] and context-aware interface [24]). Context is often referred to as information used to identify activities or events that have occurred in the smart environment. Also, some security and privacy risk models have been proposed in the literature to help users (or designers) to identify and prioritize privacy risks for a specific application [25, 26]. Other specific solutions such as cloaking area creation schemes have been used to protect the location privacy of the users [27]. However, these solutions do not consider the privacy protection for data collected or stored in the environments. Instead, they try to prevent the leakage of sensitive information during the real-time data collection. Since our work in this paper is on the privacy protection for data analysis, we will focus our discussions on the existing solutions for the secure similarity measurement.

Various procedures and protocols for testing the similarity (or homogeneity) of two or more datasets have been proposed in the literature. Private matching is a practical problem to find common data from the joint databases without revealing any private information to any party [28]. The general approach was studied by Agrawal et al. in [29] which has motivated many researchers to find efficient solutions to address the private matching problem.

In 1982, Yao introduced the first two-party computation protocol (also known as millionaires' problem) in [30]. His idea is to allow two individuals to compare their richness without revealing their wealth to each other. The protocol is secure if no parties learn extra information from the protocol execution. Since then, many secure computation protocols have been proposed to solve problems such as secure multiparty computation [13] and cooperative computation [31]. As proved by Goldreich et al. in [32], there exists a secure solution for any functionality which can be represented as a combinatorial circuit. However, the generic construction of circuit evaluation is somehow inefficient for a large number of parties because the cost for large input can be very high.

The first secure protocol to evaluate $F_{SC}$ in the semihonest setting was proposed in [33]. As shown in [34], the solution in [33] is not secure due to its potential to leak the private input of one party. Hence, another protocol with the malicious model is proposed in [34].

The most related work to our solution is the differential similarity computations proposed in [35]. Several two-party protocols have been proposed to compute exact and threshold similarities based on a specific $F_{SC}$ (e.g., scalar product and cosine similarity). In their designs, the same protocol cannot be used to facilitate another $F_{SC}$. A substantial modification is needed in order to use the same protocol to compute for other functions. Since there is no best $F_{SC}$ in the literature, we may need to consider the computation results from more than one $F_{SC}$. In this paper, we will design a solution that can be used to facilitate any $F_{SC}$ computation without modifying the existing protocol.

## 3. Technical Preliminaries

### 3.1. Homomorphic Encryption Scheme

In our protocol design, we utilize a multiplicative property from the homomorphic encryption scheme (i.e., ElGamal [36]) as our primary cryptographic tool. Let Enc_pk_(*m*) denote the encryption of *m* with the public key, pk. Given two ciphertexts Enc_pk_(*m*_1_) and Enc_pk_(*m*_2_), there exists an efficient algorithm ·_*h*_ to compute Enc_pk_(*m*_1_ · *m*_2_).

### 3.2. System Model

Our protocol consists of the following main players.

- Anonymizer *A*: a semitrusted party who helps to facilitate the computation requests.
- Requestor: a party who wants to learn the similarity between two binary datasets. The requestor will send a computation request to *A*.
- Supporter: a party who collaborates with the requestor to perform the homomorphic operations.

**Figure 1:**
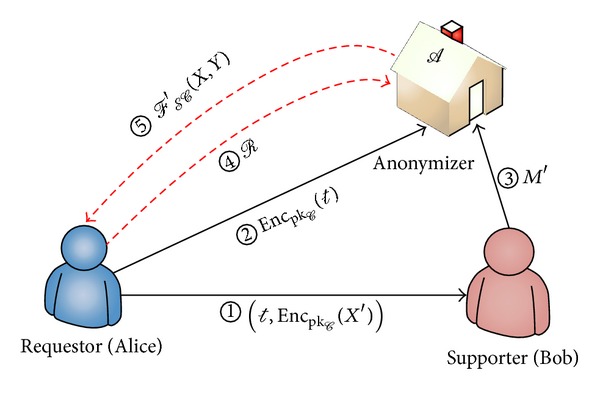
Overview of the proposed model.

Note that a supporter can also make a computation request to *A*. We can assume that the players are intelligent software agents communicating with each other in the same or from different smart environments. We can select any agent as the anonymizer if it does not collect data to be used for the computation. The interactions of players in our proposed system are shown in Figure 1.

### 3.3. Adversary Model

In general, there are two types of adversary models that can be considered: (1) the semihonest model and (2) the malicious model. In the semihonest model, all parties follow the prescribed action in the protocol but might attempt to learn extra information from the intermediate computations.

In the malicious model, a malicious party might arbitrarily deviate from the protocol for their own gain, such as performing active steps to interrupt the execution of the protocol in order to gain access to private data. In this paper, we assume that all players are semihonest parties (“honest-but-curious”). They follow the prescribed actions in the protocol but might be interested to learn some extra information from the data they received during the protocol execution or from the final output.

### 3.4. Security Model

Generally, a two-party computation problem is cast by specifying a random process that maps pairs of inputs to pairs of outputs [37]. In the setting of a two-party computation, the requestor (with input *X*) and the supporter (with input *Y*) jointly compute for the function *f*(*X*, *Y*) while preserving some security properties such as the correctness of the output and the data privacy [38].

Let Π be a protocol between the two players. Then, we can denote the requestor's output by Π_*r*_(*X*, *Y*) and the supporter's output by Π_*s*_(*X*, *Y*). Since only the client gets the output in our case, we can simply denote Π(*X*, *Y*) = Π_*C*_(*X*, *Y*). The perspective of the client and the server during the execution of protocol Π on input (*X*, *Y*) can be denoted as VIEW_*C*_^Π^(*X*, *Y*) and VIEW_*S*_^Π^(*X*, *Y*), respectively. Note that the view of each party includes their local input, their output, and their messages received from the other party. We now formally define our usage of the term privacy in our protocol (adapted from [39]) as follows.

**Definition 1 (privacy with respect to semihonest behavior).**

Let *f* : {0,1}* × {0,1}* → {0,1}* be a probabilistic polynomial-time function. One says that a two-party computation protocol Π securely computes *f* in the presence of semihonest adversaries if for every *X*, *Y* ∈ {0,1}* : Π(*X*, *Y*) = *f*(*X*, *Y*). Also, there exist probabilistic polynomial-time algorithms *S*_*C*_ and *S*_*S*_, such that

(3) $$\begin{array}{c} {\left\{S_{C}\left(X,f\left(X,Y\right)\right)\right\}}_{X,Y\in {\left\{0,1\right\}}^{*}}\overset{c}{\equiv }{\left\{{\text{VIEW}}_{C}^{\Pi }(X,Y)\right\}}_{X,Y\in {\left\{0,1\right\}}^{*}} \end{array}$$

(4) $$\begin{array}{c} {\left\{S_{S}\left(Y\right)\right\}}_{X,Y\in {\left\{0,1\right\}}^{*}}\overset{c}{\equiv }{\left\{{\text{VIEW}}_{S}^{\Pi }(X,Y),\right\}}_{X,Y\in {\left\{0,1\right\}}^{*}}, \end{array}$$

where $\overset{c}{\equiv }$ denotes computational indistinguishability according to the families of polynomial-size circuits. One refers the reader to [39] for the definition of computational indistinguishability.

Note that we can simulate each player's view by using a probabilistic polynomial-time algorithm, only given access to the party's input and output. Thus, we only need to show the existence of a simulator for each player that satisfies the requirements of (3) and (4).

### 3.5. Differential Privacy

Differential privacy is a strong notion of privacy that guarantees the privacy protection in the presence of arbitrary auxiliary information. Intuitively, it aims to limit the information leakage from the output while a small change on the inputs. The formal definition is defined as follows.

**Definition 2 (*ϵ*-differential privacy [40]).**

A randomized function *K* satisfies *ϵ*-differential privacy if, for any two neighboring datasets *D*_1_ and *D*_2_ differing on at most one element and all *S*⊆Range  (*K*),

(5) $$\begin{array}{c} \text{Pr}\left[K\left(D_{1}\right)\in S\right]\leq \exp\left(\epsilon \right)\times \text{Pr}\left[K\left(D_{2}\right)\in S\right]. \end{array}$$

**Definition 3 (global sensitivity [41]).**

The global sensitivity of a function *F* : *D* → *R* is

(6) $$\begin{array}{c} \Delta F=\underset{D_{1},D_{2}}{\max}{||F\left(D_{1}\right)-F\left(D_{2}\right)||}_{1} \end{array}$$

over all pairs of neighboring datasets *D*_1_ and *D*_2_.

**Theorem 4 (Laplacian mechanism [41]).**

For *F* : *D* → *R*, *K* achieves *ϵ*-differential if *K*(*D*) = *F*(*D*) + *Lap*(Δ*F*/*ϵ*).

The parameter *ϵ* is a small positive value which is used to control the trade-off between data privacy and data utility. A smaller value of *ϵ* will guarantee a higher privacy, but the data utility can be affected.

For $F_{SC}$ computation, we can think of *D*_1_ = (*X*, *Y*) and *D*_2_ = (*X*, *Y*′), where *Y* and *Y*′ are only differing in one element. The change of one element in *Y* will increase (or decrease) the mismatch value (*b* or *c*) by 1 and also affects the value of *a* or *d*. Therefore, Δ*F* for each $F_{SC}$ can be different depending on the formula used.

### 3.6. Notations Used 

We summarize the notations used hereafter in this paper in the Notations section.

## 4. Our Solution

In this section, we will explain the details of our computation protocol, in particular, the computation phases for each player.

### 4.1. Private Similarity Coefficients

At the preliminary phase, the semitrusted anonymizer (*A*) generates an ElGamal cryptosystem key pair (pk_*C*_, pr_*C*_) and sends the public key pk_*C*_ to all the agents. For simplicity, let us assume that there are only two agents (Alice and Bob) in the protocol. We assume that there exists a secure channel for key exchange and data transmission.

**Phase 1 .**

Alice first randomly selects a prime number *t* to replace each *x*_*i*_ ∈ *X* as follows:

(7) $$\begin{array}{c} x_{i}^{\prime }=\{\begin{array}{cc} t, & \text{if}\;\;x_{i}=1\\ t^{-1}, & \text{if}\;\;x_{i}=0.\; \end{array} \end{array}$$

Next, Alice encrypts *t* and each *x*_*i*_′ ∈ *X*′ by using pk_*C*_ (e.g., Enc_pk_*C*__(*x*_*i*_′)). Alice sends Enc_pk_*C*__(*t*) and (*t*, Enc_pk_*C*__(*X*′)) to *A* and Bob, respectively.

**Phase 2 .**

Bob replaces each *y*_*i*_ ∈ *Y* as follows:

(8) $$\begin{array}{c} y_{i}^{\prime }=\{\begin{array}{cc} t^{2}, & \text{if}\;y_{i}=1\\ t, & \text{if}\;y_{i}=0. \end{array} \end{array}$$

Next, Bob encrypts *y*_*i*_′ ∈ *Y*′ with pk_*C*_ to produce Enc_pk_*C*__(*Y*′) = {Enc_pk_*C*__(*y*_*i*_′)∣*i* = 1,2,…, *n*}. Note that the sequence of all encrypted data is the same as its sequence order in the original dataset.

**Phase 3 .**

In this phase, Bob computes Enc_pk_*C*__(*X*′*Y*′) by using the homomorphic multiplicative property. The multiplication is done in accordance with the sequence *i* of *x*_*i*_′ and *y*_*i*_′ (e.g., Enc_pk_*C*__(*x*_*i*_′)·_*h*_Enc_pk_*C*__(*y*_*i*_′) = Enc_pk_*C*__(*x*_*i*_′*y*_*i*_′)). Next, Bob randomly permutes elements in Enc_pk_*C*__(*X*′*Y*′). We assume that there exists an efficient shuffle protocol *π* which randomly changes the sequence of elements in Enc_pk_*C*__(*X*′*Y*′). For simplicity, let *M* = Enc_pk_*C*__(*X*′*Y*′) and *M*′ = *π*(*M*). Bob transmits *M*′ to *A* without revealing *M* to any party.

**Phase 4 .**

After receiving *M*′ from Bob, *A* decrypts each *m*_*i*_′∈*M*′ with its private key pr_*C*_:

(9) $$\begin{array}{c} {\text{Dec}}_{{\text{pr}}_{C}}\left({\text{Enc}}_{{\text{pk}}_{C}}\left(x_{i}^{\prime }y_{i}^{\prime }\right)\right)=x_{i}^{\prime }y_{i}^{\prime }. \end{array}$$

Next, *A* examines the decrypted values and computes the summation variables as follows:

(10) $$\begin{array}{c} x^{\prime }y^{\prime }=\{\begin{array}{cc} t^{3}, & \text{increases}\;\;a\text{ by 1}\\ t^{2}, & \text{increases}\;\;b\text{ by 1}\\ t^{1}, & \text{increases}\;\;c\text{ by 1}\\ t^{0}, & \text{increases}\;\;d\text{ by}\;\text{1.} \end{array} \end{array}$$

At the end of this phase, *A* obtains all the summation variables needed to compute $F_{SC}$.

**Phase 5 .**

Alice (or Bob) makes a request *R* to *A* to compute for a chosen $F_{SC}$ (i.e., Jaccard). The anonymizer computes $F_{SC}$(*X*, *Y*) and adds Laplacian noise Lap(Δ*F*/*ϵ*) to the computed result. At last, *A* sends $F_{SC}$′(*X*, *Y*) = $F_{SC}$(*X*, *Y*) + Lap(Δ*F*/*ϵ*) to Alice (or Bob). Note that this phase can be used to compute any $F_{SC}$ in [21] without repeating Phases 1 to 4.

We show the pseudocode for requestor, supporter, and anonymizer in Algorithms 1, 2, and 3, respectively.

### 4.2. Computing Sensitivity

In Phase 5, the anonymizer adds a Laplace noise to the computed result of the requested similarity coefficient function before it releases the mixture to the requestor. The amount of noise to be added is proportional to the sensitivity Δ*F* of the requested function. For instance, the sensitivity of the requested function is the measurement of the changes of the output (i.e., $F_{SC}$) when a small change happens in the input (*a*, *b*, *c*, or *d*).

For simplicity, we use Jaccard's index to demonstrate how to compute the sensitivity of a similarity coefficient. We denote Jaccard's index between *P* and *Q* as *J*(*P*, *Q*). Let us consider *X*, *Y*, and *Z* to be three binary datasets such that *Y* and *Z* are the same except for one bit:

(11) ΔF(Jaccard)=||J(X,Y)−J(X,Z)||=||aa+b+c−a±1a+b+c||=||±1a+b+c||.

As shown in (11), the difference between *J*(*X*, *Y*) and *J*(*X*, *Z*) is at most 1/(*a* + *b* + *c*). Therefore, the anonymizer can set Δ*F*(Jaccard) ≤ 1/(*a* + *b* + *c*).

Since the anonymizer is designed to facilitate more than one request, it needs to ensure that the noise being added will not affect the utility of the function. When the same request is received from the same (or different) requestor, a random noise should be used. This is to make sure that no party can learn the actual score for $F_{SC}$.

## 5. Analysis and Discussion

### 5.1. Correctness and Utility Analysis

The output of our protocol is correct and accurate if all parties follow the protocol faithfully. Let us assume that both the requestor and the supporter are semihonest. At Phase 3, the multiplication of *X*′ and *Y*′ will give a correct result due to the multiplicative property of the ElGamal cryptosystem. Therefore, we can ensure that the anonymizer will receive the correct outputs (*a*, *b*, *c*, and *d*) after the decryption. Note that the outputs at Phase 4 can be viewed as *f*(*X*, *Y*) = *at* ^3^ + *bt* ^2^ + *ct* ^1^ + *dt* ^0^. The coefficients for variables in *f*(*X*, *Y*) are the summation variables defined in Section 2.1.

In terms of utility, we can expect our protocol to achieve high accuracy. Our utility analysis is based on a set of similarity coefficients instead of specific function.

### 5.2. Security Analysis

To illustrate the efficacy of the security protection of our protocol in the presence of semihonest adversaries, we briefly explain how to simulate the view of each player using their respective inputs and outputs only (i.e., simulator *S*_*r*_ for the requestor and *S*_*s*_ for the supporter). If such simulation is indistinguishable from real world execution, it implies that the protocol does not reveal any extra information under semihonest model.

Let us assume that *S*_*r*_ simulates all internal coin flips of the requestor as described in our protocol. For instance, it simulates *n* ElGamal ciphertexts sent from the requestor to the supporter. Next, let us assume that *S*_*s*_ simulates all internal coin flips of the supporter as described in our protocol. This simulator simulates *n* ElGamal ciphertexts as the homomorphic multiplicative results. Based on the simulation for both parties, the computational indistinguishability for our protocol appears to hold on first inspection.

### 5.3. Privacy Analysis

In general, each player must ensure that it only releases the required data during the protocol execution. We assume that all communications in our protocol execution are via an authenticated channel, and the anonymizer will not reveal its private key to others as well. In order words, only the anonymizer can learn the summation variables after the decryption operation.

Based on the dataset Enc_pk*C*_(*X*′) computed by the requestor, the supporter is not able to distinguish which ciphertext is the encryption result of *t* or *t* ^−1^. This is because the ElGamal cryptosystem is semantically secure [42], such that the encryption of the same message will produce different ciphertexts due to randomization in the encryption process. Hence, the supporter learns nothing about *X* by knowing Enc_pk*C*_(*X*′) and *t*.

### 5.4. Comparisons with Existing Work

In this section, we will compare our protocol with the private similarity computations (*P* *SC*) proposed in [35]. In *P* *SC*, there are two types of settings that can be used to achieve the differential privacy: (1) data owners locally add noise to partially computed result (e.g., set intersection) and (2) anonymizer is responsible for inserting noise during the similarity computation. In both settings, all parties (data owners and anonymizer) must decide which $F_{SC}$ to be used in the computation. Unlike *P* *SC*, our protocol does not require the data owners to specify $F_{SC}$ before the protocol begins. Instead, the anonymizer will compute any $F_{SC}$ requests by the requestor in Phase 5 and inserts noise into the computation result to preserve the differential privacy of the private inputs.

The main limitation of *P* *SC* is its protocol design which only can be used to compute a specific $F_{SC}$ in each round of its protocol execution. All parties are required to start the protocol again even though they use the same inputs and $F_{SC}$ in the new computation. In addition, the same protocol requires substantial modifications before it can be used to facilitate other $F_{SC}$. Unlike the solution in [35], our design can be used to facilitate more than one $F_{SC}$. In particular, we allow the requestor to send multiple requests (for distinct $F_{SC}$) to the anonymizer using the same protocol. The data owners do not need to repeat computation steps from Phase 1 to Phase 4 if they use the same datasets as the inputs for the computation.

Another distinction between our protocol and *P* *SC* is the roles of each participating party. In *P* *SC*, the data owners must cooperate with each other to decrypt the homomorphically encrypted value in order to learn the computation result while the anonymizer takes part in the protocol by computing the intermediate results (e.g., set intersection). In our solution, the data owners only cooperate to compute the multiplicative operation while the anonymizer is responsible to perform the decryption operation and noise generation for each $F_{SC}$ request.

In terms of complexity, our protocol achieves a significant lower computational overhead as the *P* *SC*. Practically, running two or more protocols (using same datasets) for different $F_{SC}$ will incur high computation costs. Although the second setting in *P* *SC* can achieve the same complexity as our protocol, however, it only can be used to compute one $F_{SC}$. Note that both the basic construction of *P* *SC* and our protocol are based on the homomorphic cryptosystem.

## 6. Conclusion

Due to the advances in ubiquitous technologies and the demands of data privacy protection, a secure mechanism is required to increase the confidence of the users in the smart environments. In this paper, we have proposed a secure protocol to compute $F_{SC}$ within differential privacy model for data privacy protection in smart environments. Although our target area is a smart environment, the same solution can be applied to other related areas such as pervasive or ubiquitous computing [43] and intelligent environments [44].

In order to preserve differential privacy, the anonymizer needs to compute distinct noise for each request especially when the requestor sends the same $F_{SC}$ request. This is because the identical output may allow the adversary from learning the private dataset of the owner or noise added to the computation result. Since the same request for a specific $F_{SC}$ may output two slightly different results (due to the noise added by *A*), we can ensure that the result from our protocol execution will not compromise the privacy of any data owner. Hence, agents in smart environments can utilize our protocol to compare datasets with other entities without compromising the data privacy of the users.

## Figures and Tables

**Algorithm 1:**
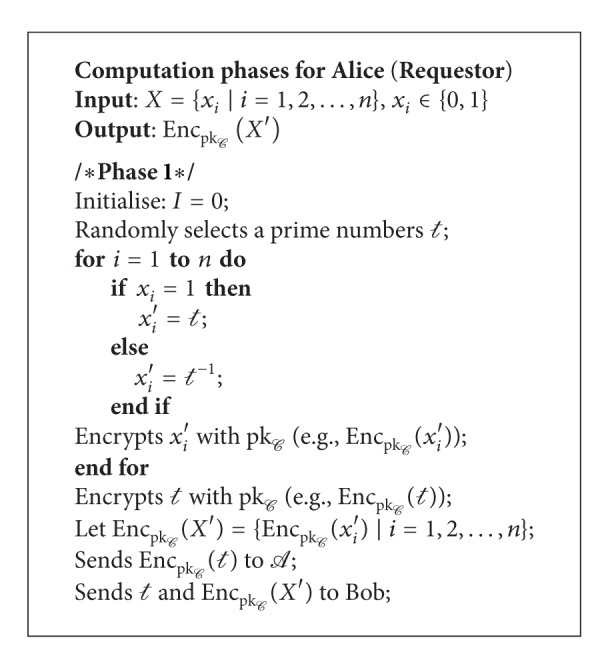
Requestor's computation phases.

**Algorithm 2:**
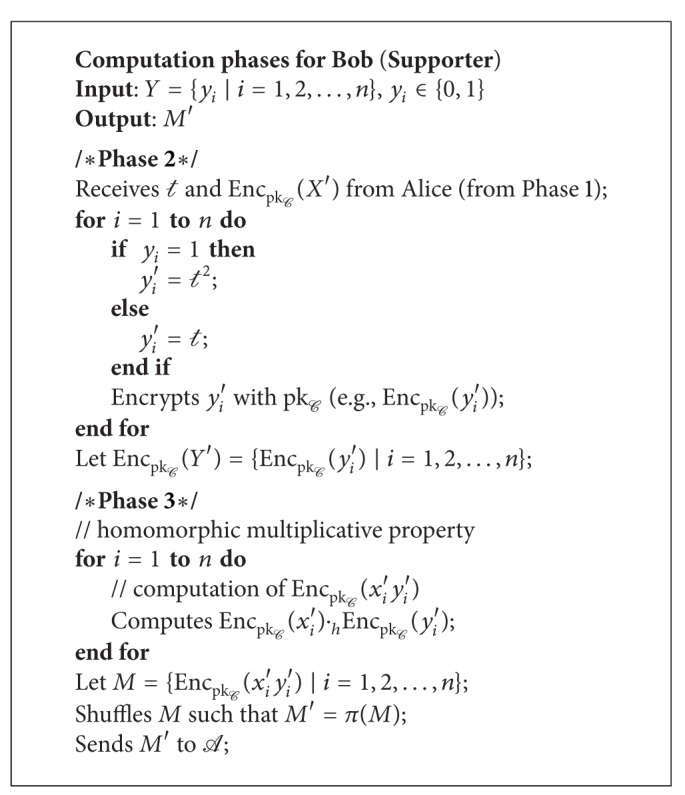
Supporter's computation phases.

**Algorithm 3:**
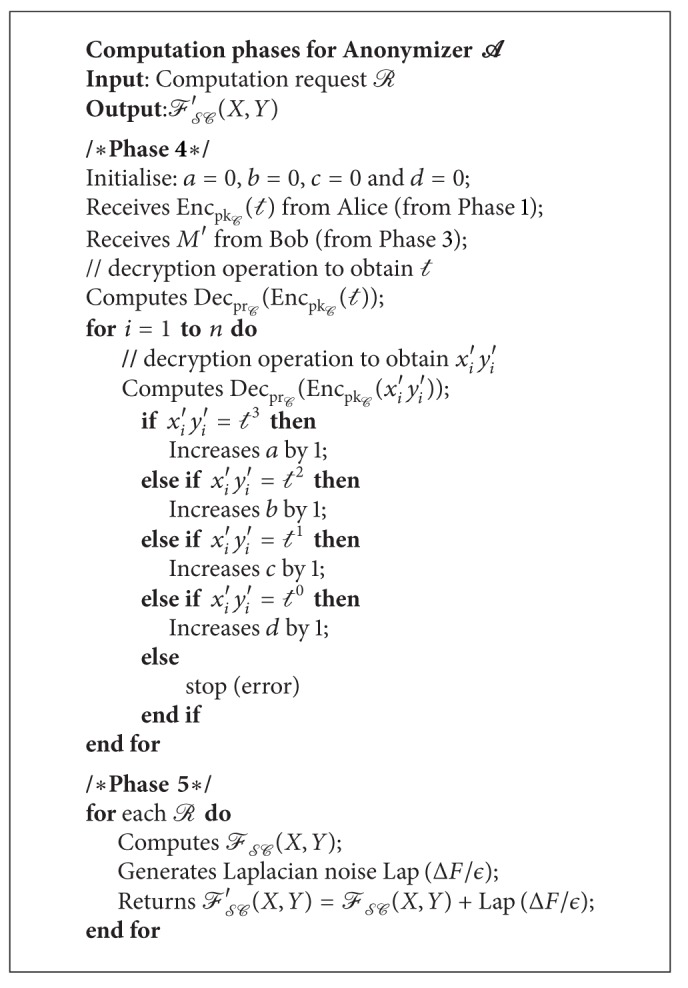
Anonymizer's computation phases.

## Notations

*X*: Private dataset from the requestor
*Y*: Private dataset from the supporter
*x*_*i*_: *i*th element of *X*
*y*_*i*_: *i*th element of *Y*
*n*: Size of the private input
*t*: Prime number chosen by anonymizer
*R*: Computation request from requestor
pk_*C*_: Public key of the anonymizer
pr_*C*_: Private key of the anonymizer
Enc_pk_*C*__(·): Encryption operation using pk_*C*_
Dec_pr_*C*__(·): Decryption operation using pr_*C*_
*π*: Shuffling protocol
*F*_*SC*_: Similarity coefficient function
Δ*F*: Sensitivity of $F_{SC}$
*F*_*SC*_′: Similarity coefficient with noise.
